# Outcome following patent ductus arteriosus ligation in premature infants: a retrospective cohort analysis

**DOI:** 10.1186/1471-2431-6-15

**Published:** 2006-05-11

**Authors:** LLeona CL Lee, Angela Tillett, Robert Tulloh, Robert Yates, Wilf Kelsall

**Affiliations:** 1 NICU, Southmead Hospital, Bristol, UK; 2 Department of Paediatrics, Colchester General Hospital, Turner Road, Colchester, UK; 3 Department of Congenital Heart Disease, Paul O'Gorman Building, Bristol Royal Hospital for Children, Upper Maudlin Street, Bristol, UK; 4 Cardiothoracic Unit, Great Ormond Street Hospital, London, UK; 5 NICU Box 226, Addenbrookes NHS Trust, Hills Road, Cambridge, CB2 2QQ, UK

## Abstract

**Background:**

The patent ductus arteriosus (PDA) is an important problem in premature infants. Surgical PDA ligation is usually only be considered when medical treatment has either failed or was contraindicated. The aims of our study were to determine the mortality and morbidity following patent ductus arteriosus ligation in premature infants, and whether prostaglandin synthetase inhibitor (PSI) use prior to ligation affects outcome.

**Methods:**

A retrospective case note review study to determine the outcome of premature infants undergoing patent ductus arteriosus ligation in one tertiary neonatal intensive care unit and two paediatric cardiothoracic centres.

**Results:**

We had follow-up data on 87 infants. Cumulative mortality rates at 7 days, 30 days and at hospital discharge were 2%, 8% and 20% respectively. The incidence of chronic lung disease, intraventricular haemorrhage, necrotising enterocolitis and retinopathy of prematurity were 77%, 39%, 26% and 28% respectively. There was no difference in mortality, incidence of chronic lung disease or duration of oxygen dependence between those who had and those who had not received a PSI prior to surgical ligation. In those who had received 2 or more courses of PSI prior to surgical ligation, there was a trend to increase in the duration of oxygen therapy and chronic lung disease, but no difference in mortality.

**Conclusion:**

This study shows that patent ductus arteriosus ligation is a relatively safe procedure (30 day survival 92%) but there is substantial late mortality and a high incidence of morbidity in the survivors. 2 or more courses of PSI prior to surgical ligation trends to increased oxygen dependence and chronic lung disease. This high risk population requires careful follow-up. A definitive prospective cohort study is lacking.

## Background

The patent ductus arteriosus (PDA) is an important problem in premature infants [1]. Left to right shunting through the PDA results in increased pulmonary blood flow and steal from the systemic circulation. These haemodynamic changes may be responsible for the co-morbid conditions associated with a PDA: prolonged ventilator dependence and chronic lung disease (CLD) [2], pulmonary haemorrhage (PH) [3], intraventricular haemorrhage (IVH) [4], necrotising enterocolitis (NEC) [5] and retinopathy of prematurity (ROP) [6]. Neonatologists may use a variety of first line strategies to close a PDA in a symptomatic preterm infant, including careful fluid administration, diuretics and prostaglandin synthetase inhibitors (PSI) such as Indomethacin or Ibuprofen. Surgical PDA ligation would usually only be considered when medical treatment had either failed or was contraindicated.

The primary aim of this study was to determine mortality after surgical PDA ligation in three centres in the United Kingdom. Secondary aims were (1) to determine the relationship between prior use of PSI on the timing of surgical ligation, duration of oxygen dependency, chronic lung disease and mortality and (2) to describe the incidence of CLD, IVH, NEC and ROP in this neonatal population.

## Methods

Infants who underwent surgical PDA ligation were identified from databases held at three hospitals. Centre A the Neonatal Intensive Care Unit (NICU) Addenbrooke's Hospital, Cambridge (1995–2000), Centre B the Department of Paediatric Cardiology, Guy's and St Thomas' Hospitals, London (1995–1999) and Centre C the Department of Paediatric Cardiology, Great Ormond Street Hospital, London (1999–2000). Infants were excluded if they were greater than 35 weeks gestation at birth, had other congenital heart disease requiring surgery or had been discharged home from the neonatal unit before surgical ligation was performed.

A retrospective case note review was performed. At the time of the study, ethics committee approval was not required. Permission to access the notes was granted by the Research and Development Department at Great Ormond Street Hospital, the Audit Lead at Guy's and St Thomas' Hospital and the Audit Department at Addenbrooke's Hospital. Pre-operative details collected included birth gestation, birth weight, and use of prostaglandin synthetase inhibitors. Operative details included age and weight at ligation. Paediatricians referring infants to centres B and C were contacted to provide follow-up information on infants discharged from the cardiothoracic centres. Postoperative details recorded were outcome, age and cause of death, duration of ventilation and total number of days of oxygen dependency. The presence of CLD (defined as oxygen dependency at 36 weeks corrected gestational age), IVH, NEC and ROP was also recorded. Statistical analysis of data was performed using the Kruskal Wallis test, the Mann Whitney U test, and Fishers exact test. Approximate 95% intervals for non-parametric data were obtained.

## Results

98 patients were identified from the three databases: 21 from centre A, 42 from centre B and 35 from centre C. Follow up data was available from 21/21 from Centre A, 35/42 from Centre B and 31/35 from Centre C. For the whole cohort, pre-operative data, duration of oxygen dependency, incidence of CLD, NEC, and IVH was available for 84 (86%) patients. Outcome survival data with information on the age and cause of death was available in 87(89%) patients. Details of PSI use were available for 82 (84%) and incidence of ROP was recorded in 79 (81%) patients.

### Demographic details

Demographic details of the infants undergoing surgical PDA ligation are shown in Table 1. There were no significant differences in birth gestation, birth weight or operation weight between the three centres. Surgical ligation tended to be performed later in centre B than in centres A and C (p = 0.05).

**Table 1:** Patient demographics of infants undergoing PDA ligation in the 3 centres – Centre A – Cambridge, Centre B – Guys and St Thomas, Centre C – Great Ormond Street. Results are expressed as median (95% confidence interval).

Demographic	Centre A n = 21	Centre B n = 35	Centre C n = 28	p value*
Birth weight (grams) Median (95% CI)	820 (649–1020)	770 (710–840)	807 (728–980)	0.67
Birth gestation (weeks) Median (95% CI)	27 (24–28)	25 (25–26)	26 (25–27)	0.32
Ligation weight (grams) Median (95% CI)	963 (781–1169)	1050 (910–1170)	950 (795–1100)	0.66
Ligation age (days) Median (95% CI)	23 (20–31)	31 (25–41)	27 (21–30)	0.05

*Kruskall Wallis test

### Mortality following PDA ligation

There were 17 deaths among the 87 patients where outcome data was available. The causes and ages of the death are shown in Table 4. The median age of death after surgery was 31 (range 6 – 134) days. For the whole cohort, postoperative mortality was 2% at 7 days, 8% at 30 days and 20% beyond day 30 up to hospital discharge. 30 day mortality was 1/21 (5%), 4/35 (11%), and 2/31 (6%) from Centres A, B and C respectively. Survival to hospital discharge was 72% in centre A, 83% in centre B and 84% in centre C. However no long term outcome data was available for 7 infants from centre B and 4 infants from centre C. Only one death was directly attributed to surgery performed in a cardiothoracic centre. This infant underwent a second thoracotomy to treat a persisting chylothorax. During this procedure the aorta was damaged and the infant subsequently died from renal failure. Ten of the deaths resulted from respiratory complications: CLD (3); pulmonary interstitial emphysema (PIE) (4); Cor pulmonale (2) and bronchiolitis (1).

### The use and effect of prostaglandin synthetase inhibitors on ligation and morbidity

Sixty-five of 82 babies were treated with PSI prior to surgery. 28 infants received one course, 33 received two or more courses and 4 infants received PSI but exact treatment details were unavailable. Seventeen infants were not treated with PSI for the following reasons: renal failure (4), pre-existing NEC (4), PH (1), thrombocytopaenia (1), deemed too old (4) and not specified (3). The median age at operation of 24 (95% CI 21–27) days after one course of PSI, was significantly lower than the age at operation after two or more courses of 31 (95% CI 25–44) days (p = 0.004). Although there was no statistical significance, the 95% confidence intervals showed a clinically relevant increase in duration of oxygen requirement after 2 or more courses of PSI. There was similarly a clinically important trend to increasing chronic lung disease although the p value was not significant. There was no difference in mortality when ligation occurred after single or multiple courses of PSI Table (2).

**Table 2:** Comparing the outcome of PDA ligation after 1 or more courses of prostaglandin synthetase inhibitors

Parameter	Ligation after 1 course of PSI (n = 28)	Ligation after >1 course of PSI (n = 33)	p value
Age at operation. Median (95% CI)	24 (21–27) days	31 (25–44) days	0.004*
Total days in oxygen. Median (95% CI)	85.5 (58–120)	119 (89–175)	0.09*
Incidence of CLD	19 (68%)	28 (85%)	0.14^¥^
Deaths	7 (25%)	8 (24%)	1.0^¥^

*Mann Whitney U test

^¥^Fisher exact test

Comparison between the group of infants who did not receive any PSI prior to ligation with those who did receive a PSI, showed no significant effect on duration of oxygen dependency, the incidence of CLD or mortality Table (3).

**Table 3:** Comparison of the outcome of PDA ligation with and without previous prostaglandin synthetase inhibitor

Parameter	PDA ligation without previous PSI (n = 17)	PDA ligation after PSI (n = 65)	p value
Total days in oxygen Median (95% CI)	83 (48–142) days	102 (78–126) days	0.20*
Incidence of CLD	13 (76%)	50 (77%)	1.00^¥^
Deaths	2 (12%)	15 (23%)	0.50^¥^

* Mann Whitney U test

^¥^Fisher exact test

**Table 4:** Cause of death and postoperative day of death following PDA ligation

Cause of Death		Postoperative days to death
Respiratory	Chronic lung disease	76, 100, 134
	Pulmonary interstitial emphysema	6, 7, 13, 17
	RSV bronchiolitis and CLD	31
	Cor pulmonale secondary to CLD	45, 47
Aspiration		67
Renal Failure		50, 56
Sepsis		9,22
Sudden infant death		47
Unknown		22

### Morbidity following PDA ligation

Morbidity outcome data was available for up to 84 infants. The incidence of chronic lung disease was 77% (65/84), intraventricular haemorrhage 39% (33/84), necrotising enterocolitis 26% (22/84) and of retinopathy of prematurity 28% (22/79).

## Discussion

The results from this three centre study show high survival rates of 98% and 92% at 7 and 30 days respectively following surgical PDA ligation. These rates are similar to previous studies that have shown that PDA ligation is a relatively safe procedure [7,8], whether it is done on a NICU [9] or after transfer to a cardio-thoracic centre [10]. In the NICU (centre A) the procedure was performed by one of two consultant adult cardio-thoracic surgeons trained in PDA ligation. In the paediatric cardiothoracic centres B and C the ligations were carried out by specialist registrars and consultant paediatric cardiothoracic surgeons. Where local expertise is available, the advantages of performing PDA ligation on NICU include: minimal disturbance of the infant; no requirement for transportation; no separation from parents and easier scheduling of the procedure.

The short-term outcome following PDA ligation was excellent, but there was a further 12% mortality between 30 days and hospital discharge. We feel that this late mortality was not due to the surgery itself but rather represented that fact that this population was already at high risk of morbidity and mortality, which was then compounded by a PDA. Respiratory complications accounted for the majority of infant deaths in this study, which is not unexpected given the overall incidence of CLD of 77%. The analysis of the data on late mortality is limited by the lack of follow up data from the cardiothoracic centres. This is a disadvantage of a retrospective study. We are unable to estimate the effect of this loss of follow up on our results. The results from centre A, for whom we have 100% follow-up, give a 72% survival to hospital discharge which may suggest that we are overestimating survival. We acknowledge the potential effect of the missing data on our results, but feel that the high late mortality we have found is important.

The ideal treatment of the PDA is subject to much debate [11]. In the United Kingdom surgical treatment is usually considered after failure or contraindication of medical treatment. Early surgical closure of a PDA without prior indomethacin treatment has not been shown to improve survival [12]. Similarly there is no significant effect on incidence of chronic lung disease, neurodevelopmental outcome or mortality between prophylactic or selective administration of indomethacin [13]. In our study population there was a great variation in practice regarding the use of PSI and the timing of surgical ligation. Our numbers were small and so we have given approximate 95% confidence intervals as well as p values to give a clearer picture. Although there was no difference in morbidity and mortality between the groups who had or had not received a PSI, on further analysis of the group who had received a PSI, it was apparent that there was a delayed operation and increasing respiratory morbidity if 2 or more courses of PSI were given. The increased respiratory morbidity may be an effect of the prolonged left to right shunting on the pulmonary vasculature and pulmonary function.

There are circumstances where the PDA is protective to the lungs – for example in the case of pulmonary hypertension. In this situation, the appropriateness of PDA ligation is dependent on the echocardiographic assessment. Our discussion of the timing of PDA ligation is limited by not having details of the echocardiographic assessments at the referring hospital. The definition of a significant PDA is however also controversial and echocardiographic and clinical criteria for diagnosis of a significant PDA vary between units.

There were not enough subjects in our study to analyse the effects of birth gestational age or birth weight on outcome. Further work would be useful since the PDA is most prevalent in extremely premature and very low birth weight infants [14,15]. In this group medical treatment with indomethacin has been shown to be less effective and may be associated with complications [16]. Earlier consideration of surgical ligation in these infants may be appropriate; PDA ligation has been demonstrated to be safe [17] and there was a significant decrease in the incidence of NEC in a study comparing prophylactic surgery with indomethacin treatment in neonates with birth weight less than 1000 g [18].

This retrospective study provides useful data on outcomes and the incidence of complications in premature infants undergoing PDA ligation. However, there are some limitations. Firstly, the study periods in the three units were not identical, but overlap. Secondly, follow-up of infants proved difficult and full information was only available for 86% of the identified cohort. Neonatal unit follow-up has been well established for some time and a complete dataset was available for infants undergoing operations on NICU. The long term follow of patients undergoing cardiothoracic procedures remains in its infancy. PDA ligation was not considered a benchmark procedure in the analysis of the central cardiac audit database (CCAD) [19]. PDA ligation is regarded as a straightforward procedure that does not require cardiac bypass. In our population however mortality is high when compared to other non-bypass procedures.

This study is also limited as we had no controls to compare associated morbidities, especially the neurodevelopmental outcomes. We can therefore only make comparisons with previously published work. Recent studies in neonatal intensive care demonstrate a lower incidence of CLD than found in this study group, varying from 29% in infants under 32 week gestation [20] to 68% in 23–28 week gestation infants [21]. The incidence of NEC in this cohort was higher than published figures of between 6–9% in those of gestational age 29 weeks or less [22] and 9–13% in those < 34weeks and < 1500 g at birth [23]. Comparative figures for IVH and ROP are less useful due to differences in classification, but a large study of infants with gestational age 25 weeks or less reports incidences of up to 16% for grade 3 IVH and above and of up to 55% for grade 3 or worse ROP [24]. The vast majority of infants in these studies [20-24] will not have required surgical closure of PDA. The high incidence of CLD, NEC, IVH and ROP in this series illustrates that this population represents one of the sickest group of infants in NICU who often require prolonged ventilation.

In summary, we feel that the long term mortality of up to 20% and the associated morbidity cannot be ignored. We would recommend that paediatricians and cardiologists consider a collaborative nationwide prospective study to look at the relationship between timing of ductal ligation and outcome in this neonatal population. Such studies should not be restricted to 30 day or 12 month survival as in the CCAD analysis. Longer term follow-up will be necessary to identify the later deaths and associated morbidity including neurodevelopmental outcome.

## Conclusion

This study shows that patent ductus arteriosus ligation is a relatively safe procedure (30 day survival 92%) but there is substantial late mortality and a high incidence of morbidity in the survivors. 2 or more courses of PSI prior to surgical ligation trends to increased oxygen dependence and chronic lung disease. This high risk population requires careful, long term follow-up. A definitive prospective cohort study is lacking.

## Abbreviations

Patent ductus arteriosus PDA; chronic lung disease CLD; necrotising enterocolitis NEC; intraventricular haemorrhage IVH; retinopathy of prematurity ROP; prostaglandin synthetase inhibitor PSI

## Competing interests

The author(s) declare that they have no competing interests.

## Authors' contributions

WK conceived of the study and participated in its design and coordination. RY and RT were the lead clinicians involved in the cardiothoracic centres. WK, LL, and AT collected and analysed the data. LL performed the statistical analysis and drafted the manuscript. All authors read and approved the final manuscript.

## Pre-publication history

The pre-publication history for this paper can be accessed here:


